# Blockade of PLD2 Ameliorates Intestinal Mucosal Inflammation of Inflammatory Bowel Disease

**DOI:** 10.1155/2016/2543070

**Published:** 2016-09-18

**Authors:** Guangxi Zhou, Lin Yu, Wenjing Yang, Wei Wu, Leilei Fang, Zhanju Liu

**Affiliations:** Department of Gastroenterology, The Shanghai Tenth People's Hospital of Tongji University, Shanghai 200072, China

## Abstract

*Background*. Inflammatory bowel diseases (IBD), including Crohn's disease (CD) and ulcerative colitis (UC), are chronically remittent and progressive inflammatory disorders. Phospholipase D2 (PLD2) is reported to be involved in the pathogenesis of several inflammatory diseases. However, the exact role of PLD2 in IBD is obscure.* Methods*. PLD2 expression was determined in peripheral blood cells and inflamed mucosa from patients with IBD by qRT-PCR. Colonic biopsies were also obtained from CD patients before and after infliximab (IFX) treatment to examine PLD2 expression. PLD2 selective inhibitor (CAY10594) was administrated daily by oral gavage in DSS-induced colitis mice. Bone marrow neutrophils from colitis mice were harvested to examine the migration using Transwell plate.* Results*. PLD2 was found to be significantly increased in peripheral blood cells and inflamed mucosa in patients with active IBD. Treatment with IFX could significantly decrease PLD2 expression in intestinal mucosa in patients with CD. Moreover, blockade of PLD2 with CAY10594 could markedly ameliorate DSS-induced colitis in mice and promote neutrophil migration.* Conclusions*. PLD2 plays a critical role in the pathogenesis of IBD. Blockade of PLD2 may serve as a new therapeutic approach for treatment of IBD.

## 1. Introduction

Inflammatory bowel diseases (IBD), comprising of Crohn's disease (CD) and ulcerative colitis (UC), are chronically remittent and progressive inflammatory disorders occurring in gastrointestinal tract [1–3], characterized by intestinal mucosal inflammation culminating in abdominal pain, recurrent diarrhea, blood stools, and loss of body weight [4, 5]. Although the underlying etiology and pathology of IBD remain elusive, accumulating evidence has demonstrated that the pathogenesis of IBD is the result of abnormal immune response to intestinal microbiota in genetically susceptible individuals [6–8]. During acute inflammation, large numbers of leukocytes infiltrate into inflamed mucosa of IBD, and these leukocytes, as well as intestinal stromal and epithelial cells, produce large amounts of proinflammatory cytokines, which further contribute to inflammatory damage in gut mucosa [9]. CD4^+^ T cells, an important component of adaptive immune system, have been reported to play a critical role in the pathogenesis of IBD. Studies have implicated that T helper (Th) 1-related cytokines, such as interferon- (IFN-) *γ* and tumor necrosis factor (TNF-) *α*, Th17 associated cytokines, such as interleukin- (IL-) 17A, IL-6, and IL-23, and Th2 related cytokines such as IL-4, IL-5, and IL-13 are involved in the induction and development of IBD [10]. Neutrophils, the most abundant leukocyte population, are also indispensable in protecting the host from the invading of microbial pathogens [11]. They are endowed with the several capacities of antimicrobial functions, such as phagocytosis, formation of neutrophil extracellular trap (NET), degranulation, and release of reactive oxygen species (ROS) [12]. Moreover, neutrophils can also facilitate epithelial repairing and mucosal healing by releasing cytokines and chemokines necessary for the resolution of mucosal inflammation [12, 13].

Phospholipase D2 (PLD2), a lipid-signaling enzyme, is a member of PLD family and can catalyze the hydrolysis of phosphatidylcholine (PC) to produce the signaling molecule phosphatidic acid (PA) [14]. PLD2 and PA can interact with several types of phosphatases, kinases, proteins and phospholipases, and further mediate multiple cellular functions, including activation, proliferation, apoptosis, and migration [14–17]. Evidence has shown that PLD2 participates in the pathogenesis of several diseases, such as pathological angiogenesis, sepsis, asthma, Alzheimer's disease, and cancer [18–21]. It has been found to be involved in the enhancement of basal permeability through regulating cytoskeleton reorganization and occludin expression [22]. Inhibition of PLD2 could promote colon cancer cell apoptosis through downregulating PI3K-AKT signaling pathway and play a protective role in colonic cancer [23]. Moreover, PLD2 also induces the aggravation and drives mortality in sepsis by inhibiting the formation of neutrophil extracellular trap and reducing the expression of CXCR2 [18]. These data suggest that PLD2 was involved in the development of inflammation and colorectal cancer; however, whether PLD2 participates in the pathogenesis of IBD remains unknown.

In this study, we found that PLD2 was highly expressed in peripheral blood cells and inflamed mucosa in patients with active IBD. Stimulation with TNF-*α* could markedly enhance PLD2 expression in neutrophils, and treatment with IFX could reverse the increased expression of PLD2. Moreover, inhibition of PLD2 with CAY10694 could significantly ameliorate DSS-induced intestinal colitis in mice. Inhibition of PLD2 could also promote neutrophil migration through upregulating CXCR2 expression. Therefore, our data suggest that blockade of PLD2 ameliorates intestinal mucosal inflammation and that PLD2 could be a therapeutic target for treatment of IBD.

## 2. Materials and Methods

### 2.1. Patients

All peripheral blood and colonic biopsies samples were collected from IBD patients from July 2014 to October 2015 at the Department of Gastroenterology, the Shanghai Tenth People's Hospital of Tongji University (Shanghai, China). Peripheral blood samples were obtained from patients with active CD (A-CD, *n* = 25), patients with CD in remission (R-CD, *n* = 19), patients with active UC (A-UC, *n* = 20), patients with UC in remission (R-UC, *n* = 21), and healthy controls (*n* = 28). Colonic biopsy samples were collected from patients with A-CD (*n* = 21), R-CD (*n* = 27), A-UC (*n* = 26), R-UC (*n* = 26), and HC (*n* = 18) during colonoscopy. The final diagnoses for CD or UC were based on clinical characteristics, radiological and endoscopic examination, and histological findings (see Supplementary Table 1 in Supplementary Material available online at http://dx.doi.org/10.1155/2016/2543070) [24]. International standard criteria such as Crohn's disease activity index (CDAI) and Mayo scores were used to assess the disease severity in patients with CD and UC, respectively [25, 26]. This study was approved by the Institutional Review Board for Clinical Research of the Shanghai Tenth People's Hospital of Tongji University. Written informed consent was also obtained from all subjects before study.

### 2.2. Anti-TNF mAb Treatment in Patients with Active CD

Seventeen patients were diagnosed as active CD according to a CDAI score ≥ 150 points and treated with anti-TNF mAb (5 mg/kg, infliximab (IFX); Cilag AG, Schaffhausen, Switzerland) at weeks 0, 2, and 6 as described previously [27]. All patients were monitored weekly during the follow-up examination, and colonic biopsies were collected at weeks 0 and 12 after the first infusion. The efficacy of IFX treatment was assessed according to CDAI and mucosal healing by endoscopy as described previously [27]. Clinical remission was defined as a CDAI score of <150 points, and clinical response as a decrease of CDAI score ≥ 70 points at the evaluation time point in comparison with the baseline index.

### 2.3. Mucosal Biopsy Culture* In Vitro*

Colonic biopsies were obtained from patients with A-CD (*n* = 17) during endoscopic examination and cultured* ex vivo* (2 biopsy samples/well) in 1 mL RPMI 1640 medium in the presence of IFX or control human IgG (HIg) (both at 50 *μ*g/mL) at 37°C in 5% CO2 humidified air for 24 h. RNA was then extracted from cultured mucosal tissues and used to the examination of PLD2 by qRT-PCR.

### 2.4. Mice

Specific pathogen-free C57BL/6 mice were purchased from the Shanghai SLAC Laboratory Animal Co. Ltd. (Shanghai, China). Mice were raised under specific pathogen-free conditions with filtered air and allowed free access to sterile water and autoclaved food. Mice used in experiment were at 8–10 weeks of age and 20–25 g of weight. Animal studies were reviewed and approved by the Institutional Animal Care and Use Committee of Tongji University.

### 2.5. Establishment of DSS-Induced Colitis Model in Mice

DSS-induced colitis model was established using a method described previously [28]. Briefly, two groups of C57BL/6 (10 mice per group) were given 2.5% DSS in the drinking water for continuous seven days, and at 8th day, all the mice were given sterile water for another three days. One group of mice was administered with PLD2 selective inhibitor (CAY10694, Santa Cruz Biotechnology, 4 mg/kg) daily by oral gavage, and another group of mice were administered with PBS as controls. Other two groups of mice (10 mice per group) were given sterile water for ten continuous days as negative controls. During the observation of 10 days, characteristics of acute colitis were observed daily, including diarrhea, bloody stools, body weight, and survival rates. At the 10th day, all the mice were sacrificed; colonic tissues were obtained from mice. A small part of colon (0.5 cm) was fixed in 10% paraformaldehyde overnight used for H&E staining, and another small part of colon (0.5–1.0 cm) was used for RNA extraction and qRT-PCR analysis. Furthermore, bone marrow cells of mice were also isolated after red blood cell lysis. Neutrophils were then isolated from bone marrow of mice using neutrophil isolation kit (Miltenyi Biotec, order: 130097658) and used for flow cytometry analysis and migration capacity analysis by Transwell plates (MultiScreen, 5 *μ*m)* in vitro*.

### 2.6. Quantitative Real-Time Polymerase Chain Reaction (qRT-PCR)

Total RNA was extracted from the fresh-frozen biopsies or mouse colonic tissues, and the quantity and quality were assessed using a NanoVue spectrophotometer (GE Healthcare, Piscataway, NJ, USA), with a 260/280 ratio between 1.8 and 2.0. The complementary DNA (cDNA) was synthesized with 5x All-In-One RT MasterMix (abm) according to the manufacturer's instructions. Reverse transcription-PCR reactions were performed using the following conditions: 25°C for 10 min and 42°C for 15 min, followed by 85°C for 5 min. The synthesized cDNA was stored at −20°C. qRT-PCR was performed using SYBR green methodology according to the following conditions: 95°C for 1 min, followed by 40 cycles at 95°C for 15 s and 60°C for 30 s with 40 cycles. All samples for qRT-PCR analysis were performed in triplicate wells. All the primers were synthesized from ShengGong BioTeck (Shanghai, China) and GAPDH was used as the endogenous reference gene (Table 1). The relative levels of target gene expression were calculated as a ratio relative to the GAPDH reference. qRT-PCR analysis was carried out using the 2^−ΔΔCt^ method [29].

### 2.7. Immunohistochemistry

Immunohistochemistry was performed on 5-*μ*m-thick sections from fresh-frozen biopsies from IBD patients and healthy controls. Sections were air-dried overnight, fixed in acetone for 10 min, and rinsed in phosphate-buffered saline (PBS) for 5 min. After incubation with EnVision FLEX Peroxidase-Blocking Reagent for 10 min, these sections were incubated with rabbit anti-human PLD2 polyclonal antibody (Abcam, dilution 1 : 100) at 4°C overnight. After washing in PBS, the sections were incubated for 60 min with HRP-conjugated goat anti-rabbit or goat anti-mouse IgG (dilution 1 : 400) at room temperature. The colour reaction was developed with 3,3′-diaminobenzidine and the sections were counterstained with haematoxylin. As negative controls, sections were treated with PBS instead of primary antibody. To determine the proportion of positive cells, five fields of intestinal mucosa were selected randomly at high power (×400) [29].

### 2.8. Statistical Analysis

Data were expressed as mean ± SEM and analyzed using SPSS statistics version 14.0 (SPSS, Chicago, IL, USA). Statistical comparisons were performed using an unpaired two-tailed Student's *t*-test or one-way analysis of variance (ANOVA). Paired *t*-test was performed to analyze the statistics before and after IFX treatment. ^*∗*^ *p* < 0.05 was considered statistically significant, ^*∗∗*^ *p* < 0.01 was considered obviously statistically significant, and ^*∗∗∗*^ *p* < 0.001 was considered very obviously statistically significant.

## 3. Results

### 3.1. PLD2 Is Highly Expressed in Peripheral Blood Cells and Inflamed Mucosa in Patients with Active IBD

**Figure 1:**
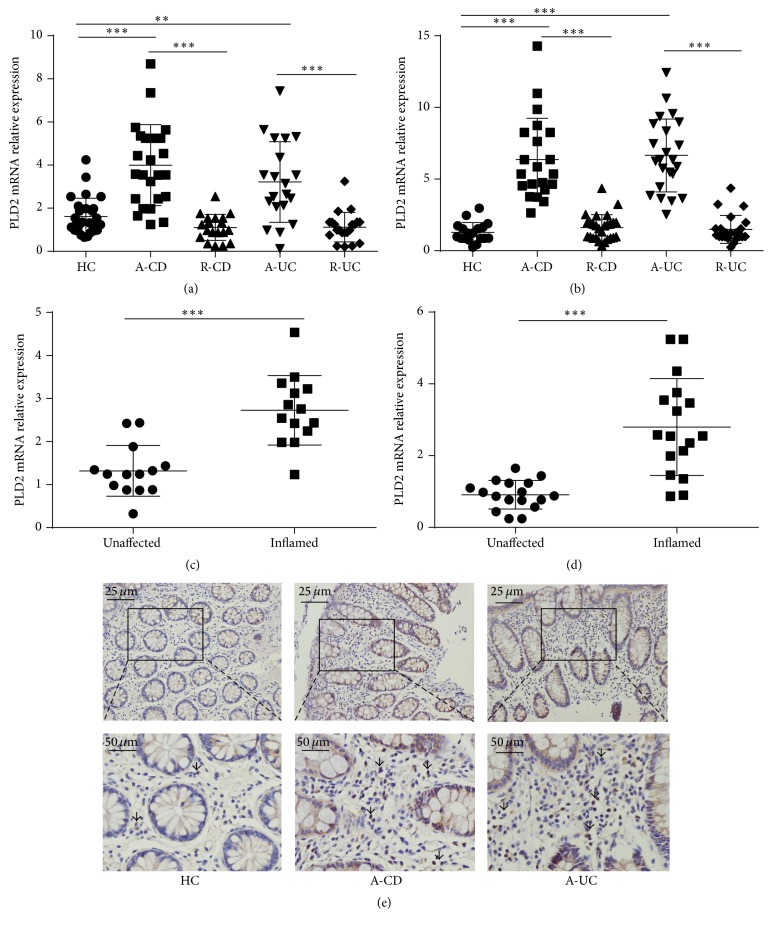
PLD2 is highly expressed in patients with active IBD. (a) Peripheral blood samples were collected from patients with active CD (A-CD, *n* = 25), patients with CD in remission (R-CD, *n* = 19), patients with active UC (A-UC, *n* = 20), patients with UC in remission (R-UC, *n* = 21), and healthy controls (*n* = 28). Expression of PLD2 mRNA was detected by qRT-PCR. (b) Colonic biopsies were collected from patients with A-CD (*n* = 21), R-CD (*n* = 27), A-UC (*n* = 26), R-UC (*n* = 26), and HC (*n* = 18). Expression of PLD2 mRNA was examined by qRT-PCR. Gene expression was normalized to GAPDH in each group. ^*∗∗*^ *p* < 0.01 and ^*∗∗∗*^ *p* < 0.001 versus HC. ((c) and (d)) Expression of PLD2 mRNA in inflamed and healthy intestinal mucosa from the same patients with A-CD ((c) *n* = 14) and A-UC ((d) *n* = 17) was examined by qRT-PCR. Gene expression was normalized to GAPDH in each group. ^*∗∗*^ *p* < 0.01 and ^*∗∗∗*^ *p* < 0.001 versus unaffected mucosa. (e) Representative images of immunohistochemical staining of PLD2 in inflamed colon from patients with A-CD, A-UC, and normal colonic mucosa from HC. Original magnification ×200 (top) and original magnification ×400 (bottom).

Previous work has demonstrated that PLD2 participates in the pathogenesis of sepsis and chronic asthma [18, 21]; we hypothesized that PLD2 may also involve the induction and development of IBD. Thus, peripheral blood and inflamed mucosa were collected from patients with active IBD and healthy controls, and we found that PLD2 expression was significantly increased in peripheral blood cells and inflamed mucosa in A-CD and A-UC patients compared with healthy controls. However, there was no significant difference between patients with R-CD or R-UC and healthy controls. No statistical difference was observed between CD and UC groups (Figures 1(a) and 1(b)). Furthermore, we compared PLD2 expression in inflamed and unaffected mucosa from the same IBD patients and found that PLD2 expression was markedly more increased in inflamed mucosa than that in unaffected controls (Figures 1(c) and 1(d)). Immunohistochemistry staining showed that a percentage of PLD2 positive cells were significantly increased in lamina propria in inflamed mucosa from patients with CD or UC compared with healthy controls (Figure 1(e)).

**Figure 2:**
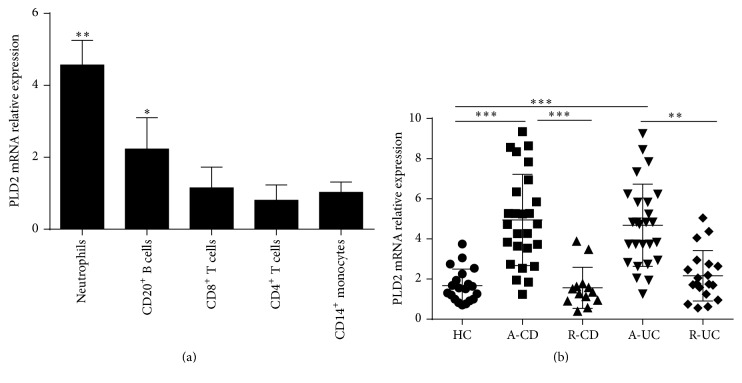
PLD2 is mainly expressed in neutrophils. (a) Expression of PLD2 in different subsets of immune cells. Peripheral neutrophils, CD20^+^ B cells, CD8^+^ T cells, CD4^+^ T cells, and CD14^+^ monocytes (1 × 10^6^) were isolated from healthy donors (*n* = 10), and expression of PLD2 was detected by qRT-PCR. ^*∗*^ *p* < 0.05, ^*∗∗*^ *p* < 0.01, and ^*∗∗∗*^ *p* < 0.001 versus CD14^+^ monocytes. (b) Neutrophils were isolated from peripheral blood of patients with A-CD (*n* = 26), R-CD (*n* = 23), A-UC (*n* = 26), R-UC (*n* = 19), and HC (*n* = 20). Expression of PLD2 mRNA was examined by qRT-PCR. ^*∗*^ *p* < 0.05, ^*∗∗*^ *p* < 0.01, and ^*∗∗∗*^ *p* < 0.001 versus HC. Gene expression was normalized to GAPDH in each group.

To determine phenotypic expression of PLD2 in different subsets of cells, we isolated neutrophils, CD4^+^ T cells, CD8^+^ T cell, CD14^+^ monocytes, and CD20^+^ B cells from healthy donors, and expression of PLD2 was analyzed by qRT-PCR.  Figure 2(a) shows that PLD2 was mainly expressed in neutrophils. Moreover, we analyzed percentage and absolute numbers of neutrophils in peripheral blood from patients with IBD and observed that percentage and absolute numbers of neutrophils were significantly increased (Supplementary Figure 1). CD66b was a specific marker expressed in neutrophils, and immunohistochemical staining revealed that CD66b^+^ cells were markedly increased in inflamed mucosa from patients with IBD compared with those in HC (Supplementary Figure 2). We then isolated neutrophils from peripheral blood of patients with IBD and found that PLD2 mRNA expression was highly increased in peripheral neutrophils from IBD patients compared with HC (Figure 2(b)). Therefore, our data indicate that PLD2 is highly expressed in peripheral neutrophils and inflamed mucosa of IBD and that it may play an important role in the pathogenesis of IBD.

### 3.2. TNF-*α* Markedly Upregulates PLD2 Expression

**Figure 3:**
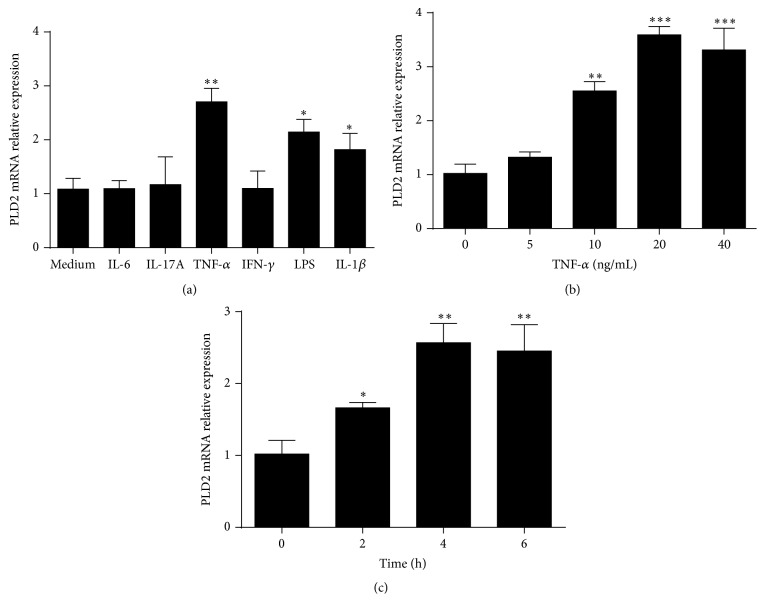
TNF-*α* upregulates PLD2 expression in neutrophils. (a) Neutrophils isolated from healthy donors (*n* = 10) were stimulated with IL-6 (10 ng/mL), IL-17A (10 ng/mL), TNF-*α* (10 ng/mL), IFN-*γ* (10 ng/mL), LPS (10 ng/mL), and IL-1*β* (10 ng/mL), respectively, for 4 h. Expression of PLD2 mRNA was detected by qRT-PCR. ^*∗*^ *p* < 0.05, ^*∗∗*^ *p* < 0.001, and ^*∗∗∗*^ *p* < 0.01 versus medium alone. (b) Neutrophils isolated from healthy donors (*n* = 10) were stimulated* in vitro* with different concentrations of TNF-*α* as indicated for 4 h; expression of PLD2 was detected by qRT-PCR. ^*∗*^ *p* < 0.05,  ^*∗∗*^ *p* < 0.01, and ^*∗∗∗*^ *p* < 0.001 versus that under culture in medium alone. (c) Neutrophils isolated from healthy donors (*n* = 10) were stimulated with TNF-*α* (10 ng/mL) for 2, 4, and 6 h, and expression of PLD2 was detected by qRT-PCR. ^*∗*^ *p* < 0.05, ^*∗∗*^ *p* < 0.01, and ^*∗∗∗*^ *p* < 0.001 versus that at the beginning of stimulation. Gene expression was normalized to GAPDH in each group.

**Figure 4:**
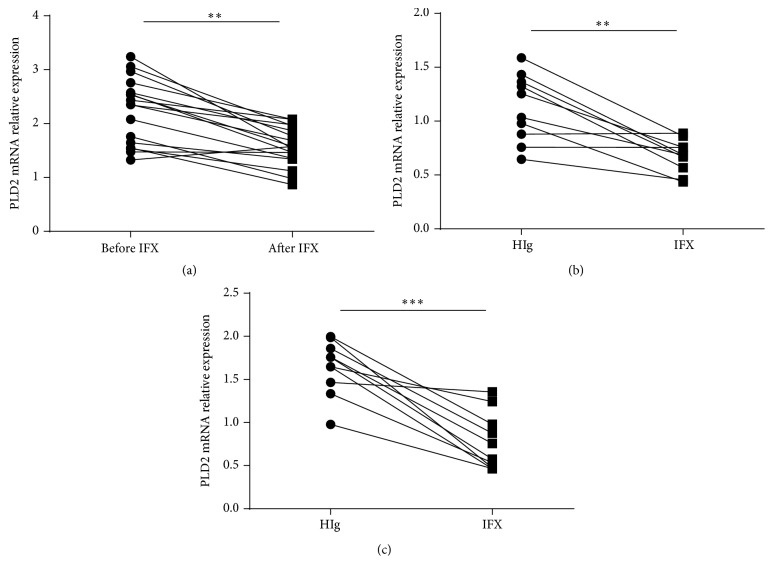
Anti-TNF therapy downregulates PLD2 expression. (a) Patients with A-CD (*n* = 17) were treated with anti-TNF mAb (IFX, 5 mg/kg) as indicated. Intestinal mucosal biopsies were collected before and at week 12 after the first infusion. Expression of PLD2 mRNA in intestinal mucosa was detected by qRT-PCR. ^*∗∗*^ *p* < 0.01 versus that before IFX treatment. ((b) and (c)) Fresh colonic biopsies were harvested from inflamed mucosa in patients with active CD ((b) *n* = 10) or active UC ((c) *n* = 10) and cultured* in vitro* with IFX or control human IgG (HIg) (50 *μ*g/mL) for 24 h. Expression of PLD2 mRNA was detected by qRT-PCR. ^*∗∗*^ *p* < 0.01 and ^*∗∗∗*^ *p* < 0.001 versus controls.

After confirming that PLD2 was increased in inflamed mucosa in patients with active IBD, we then investigated the mechanisms involved in increased expression of PLD2. Since accumulating evidences have reported that a variety of cytokines (e.g., TNF-*α*, IL-6, IL-17A, IFN-*γ*, and IL-1*β*) participate in the pathogenesis of IBD [29–32], we investigated whether these proinflammatory cytokines participated in the upregulation of PLD2 expression. Peripheral blood neutrophils were isolated from healthy donors and stimulated with IL-6, IL-17A, TNF-*α*, LPS, IFN-*γ*, and IL-1*β*, respectively, for 4 h* in vitro*. As shown in Figure 3(a), TNF-*α* greatly enhanced PLD2 expression in human peripheral neutrophils, and LPS and IL-1*β* could modestly enhance PLD2 expression. Furthermore, neutrophils were stimulated with TNF-*α* at different concentrations for different time points, and we found that TNF-*α* could upregulate PLD2 expression in a dose- and time-dependent manner (Figures 3(b) and 3(c)). Previous work has confirmed that anti-TNF-*α* treatment could ameliorate intestinal mucosal inflammation in patients with active IBD [33, 34]. Therefore, we collected colonic biopsies from patients with CD before and after treatment. Patients with active CD were also treated with IFX at weeks 0, 2, and 6 as described previously [27], and colonic biopsies were obtained from 17 patients who achieved clinical remission. PLD2 expression was found to be markedly decreased after IFX treatment (Figure 4(a)), while there was no difference in PLD2 expression in intestinal mucosa from CD patients who failed in IFX therapy. We further investigated whether IFX treatment could decrease PLD2 expression in colonic mucosa* ex vivo*. Freshly inflamed colonic tissues were collected from patients with A-CD and A-UC and cultured* in vitro* under stimulation with either IFX or control human IgG (HIg) for 24 h. As shown in Figures 4(b) and 4(c), expression of PLD2 was also markedly downregulated after IFX treatment compared with controls. Taken together, these data indicate that expression of PLD2 is increased in patients with active IBD, and anti-TNF treatment could downregulate PLD2 expression in intestinal mucosa.

### 3.3. Blockade of PLD2 Ameliorates DSS-Induced Colitis in Mice

**Figure 5:**
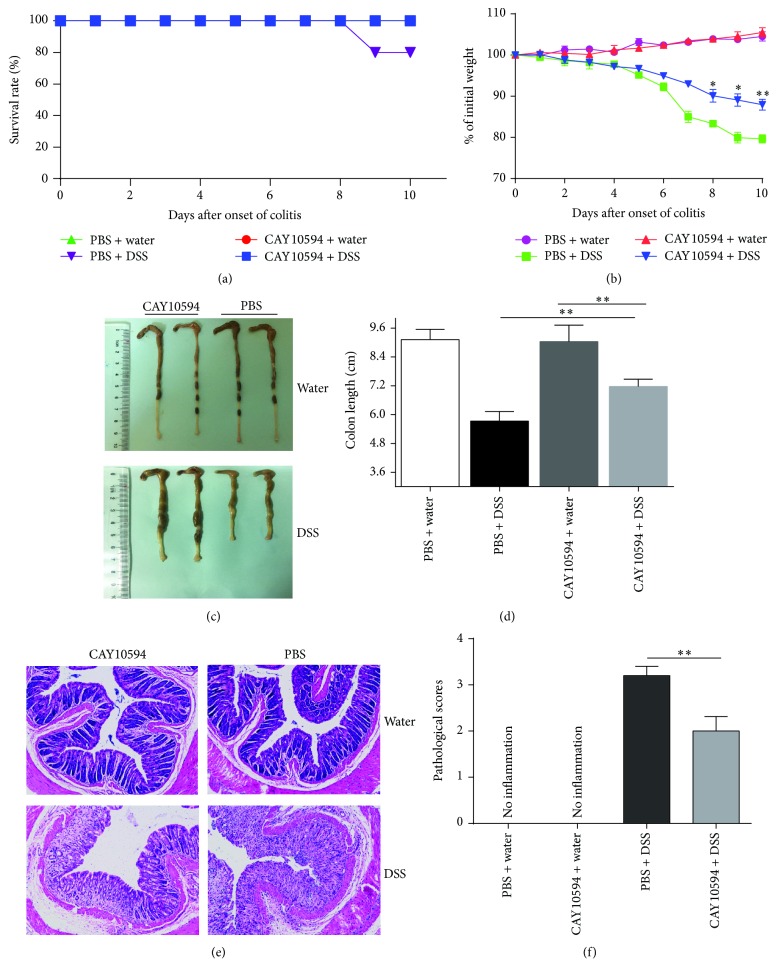
Blockade of PLD2 alleviates DSS-induced colitis in mice. DSS-induced colitis in C57BL/6 mice was induced as indicated. Two groups of DSS-exposed mice (*n* = 10) were treated with PLD2 inhibitor (CAY10594, 4 mg/Kg) or PBS as controls daily by oral gavage. Two groups of none DSS-exposed mice (*n* = 10) were also treated with CAY10594 or PBS as negative controls. (a) The survival rates of mice after DSS exposure over 10 days. (b) The changes of body weight were observed and expressed as a percentage of initial body weight at the start of experiments during 10-day observation. (c) Gross morphology of colonic tissues on day 10 after DSS induction. (d) The statistical length of colons in different groups. ^*∗*^ *p* < 0.05 and ^*∗∗*^ *p* < 0.01 versus controls. (e) Representative H&E staining images of colonic sections (×200). (f) The changes in pathological scores from colonic sections were calculated as indicated. ^*∗*^ *p* < 0.05 and ^*∗∗*^ *p* < 0.01 versus controls.

**Figure 6:**
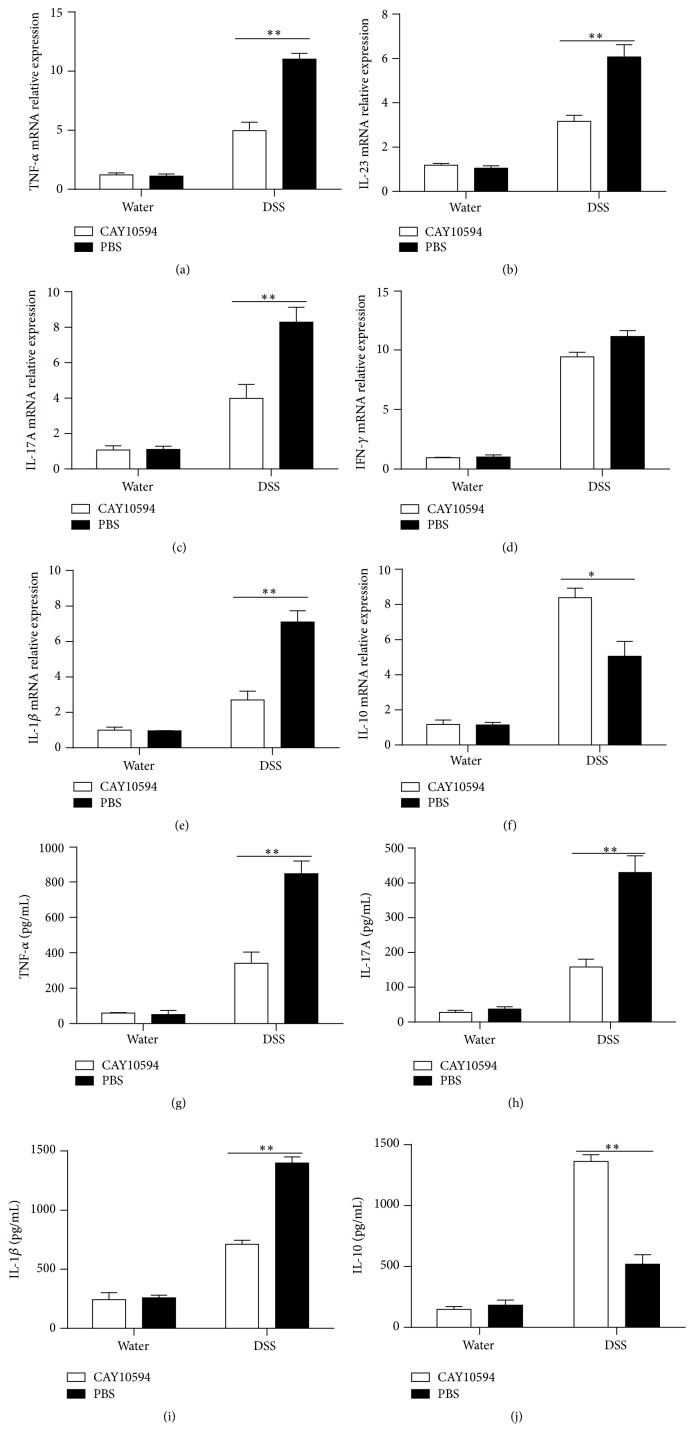
Cytokines profiles in colonic tissues from DSS-induced colitis mice after PLD2 inhibition. ((a)–(f)) Colonic tissues were obtained from mice on day 10 after DSS-induced colitis, and total RNA was extracted to detect mRNA levels of various cytokines by qRT-PCR. ^*∗*^ *p* < 0.05 and ^*∗∗*^ *p* < 0.01 versus controls. ((g)–(j)) Colonic tissues (0.01 g/sample) from mice on day 10 after DSS-induced colitis were cultured* ex vivo* at 37°C for 24 h; the supernatants were then collected for detection of TNF-*α*, IL-17A, IL-1*β*, and IL-10 by ELISA. ^*∗*^ *p* < 0.05 and ^*∗∗*^ *p* < 0.01 versus controls.

To further investigate the potential role of PLD2 in the pathogenesis of intestinal mucosal inflammation, acute colitis was induced in C57BL/6 mice by 2.5% DSS as described in Materials and Methods. PLD2 selective inhibitor CAY10594 (4 mg/Kg) was then administrated daily by oral gavage as indicated [18], and PBS as medium was also administrated daily as controls. Another two groups of mice were administrated CAY10694 or PBS daily as mentioned above without DSS exposure to serve as negative controls. Clinical characteristics of colitis, such as diarrhea, bloody stools, body weight, and survival rate, were observed daily. On day 10, all the mice were sacrificed, and colonic tissues and bone marrow cell were obtained for further study. As shown in Supplementary Figures 3(A) and 3(B), expression of PLD2 was observed to be markedly increased in inflamed mucosa and bone marrow-derived neutrophils in mice with DSS-induced acute colitis. Interestingly, administration of CAY10594 markedly ameliorated intestinal mucosal inflammation, characterized by higher survival rates, slighter decrease of body weight, less or even no bloody stools, and lower levels of pathological scores compared with controls (Figure 5). RNA was extracted from colonic tissue to detect cytokine expression, and expression of proinflammatory cytokines, such as TNF-*α*, IL-6, IL-23, and IL-1*β*, was found to be markedly decreased after blockade of PLD2 in DSS-induced colitis, whereas anti-inflammatory cytokine (e.g., IL-10) was significantly increased after inhibition of PLD2 (Figures 6(a)–6(f)). Moreover, fresh colonic samples were also obtained and cultured* in vitro* for 24 h; the supernatants were collected to detect cytokines by ELISA. As shown in Figures 6(g)–6(j), the levels of proinflammatory cytokines (e.g., IL-17A, TNF-*α*, and IL-1*β*) were found to be decreased, while anti-inflammatory cytokine (e.g., IL-10) was found to be increased after blockade of PLD2, suggesting that blockade of PLD2 could ameliorate intestinal mucosal inflammation.

### 3.4. Blockade of PLD2 Improves Neutrophil Migration

**Figure 7:**
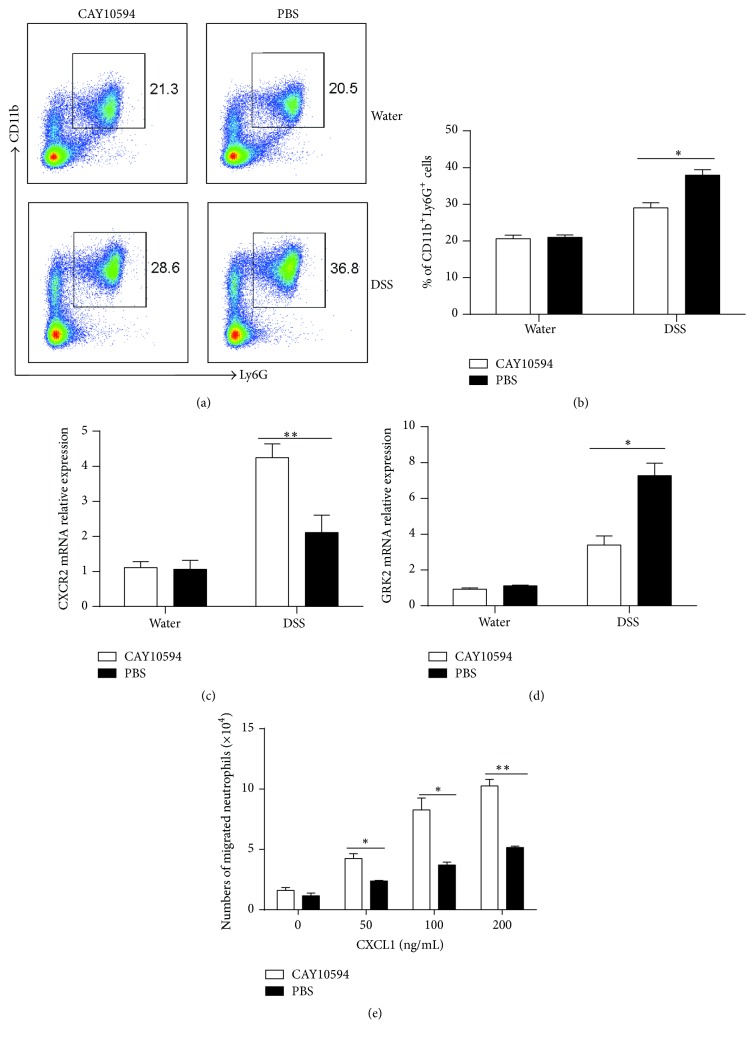
Inhibition of PLD2 promotes neutrophil migration. (a) Bone marrow cells were isolated from DSS-induced mice on day 10, and expression of Ly6G and CD11b was analyzed by flow cytometry. (b) Percentages of Ly6G^+^CD11b^+^ neutrophils were calculated. ^*∗*^ *p* < 0.05 and ^*∗∗*^ *p* < 0.01 versus controls. ((c) and (d)) Neutrophils were isolated from the bone marrow of mice; expression of CXCR2 (c) and GRK2 (d) was examined by qRT-PCR. ^*∗*^ *p* < 0.05 and ^*∗∗*^ *p* < 0.01 versus controls. (e) Neutrophils were isolated from the bone marrow of mice on day 10 after DSS-induced colitis and stimulated* in vitro* with PLD2 inhibitor (CAY10594, 10 *μ*m) for 30 min; neutrophil migration was then analyzed by a Transwell plate (5 *μ*m) under the stimulation with CXCL1 (0, 50, 100, 200 ng/mL) for 30 min. ^*∗*^ *p* < 0.05 and ^*∗∗*^ *p* < 0.01 versus controls.

To determine how PLD2 regulated intestinal mucosal inflammation, we isolated bone marrow cells from mice with DSS-induced colitis. As shown in Figures 7(a) and 7(b), the percentage of neutrophils in bone marrow was found to be significantly decreased after blockade of PLD2 in DSS-induced colitis mice, indicating that PLD2 was involved in the capacity of neutrophils migration. Previous data has shown that CXCR2, a chemokine receptor, is mainly expressed in neutrophils and mediates neutrophil mobilization to peripheral blood and inflammatory sites, which is regulated by G protein-coupled receptor kinase (GRK2) [35, 36]. We isolated neutrophils from bone marrow of mice with DSS-induced acute colitis and found that expression of CXCR2 was significantly increased in inflamed colon of DSS-induced colitis mice after blockade of PLD2 and that GRK2 expression was markedly decreased after PLD2 inhibition compared with WT controls (Figures 7(c) and 7(d)). To further determine the role of PLD2, we isolated neutrophils from bone marrow of wild-type mice with DSS-induced colitis and analyzed neutrophil migration in Transwell plate* in vitro*. After inhibition of PLD2* in vitro*, the capacity of neutrophil migration was significantly upregulated (Figure 7(e)). Taken together, these data suggest that inhibition of PLD2 may enhance neutrophil migration capacity, promote its mobilization to inflammatory intestinal mucosa, and further ameliorate mucosal inflammation.

## 4. Discussion

The main characteristics of IBD are multiple inflammatory responses associated with mucosal damage, increased epithelial permeability, bacterial invasion, and massive recruitment of neutrophils [8]. Neutrophils are one of major immune cells involved in the pathogenesis of IBD. In this study, we demonstrated that PLD2 was mainly expressed in neutrophils and that TNF-*α* could upregulate PLD2 expression. Importantly, blockade of PLD2 markedly ameliorated intestinal mucosal inflammation through enhancing neutrophils migration. Therefore, our data suggest that PLD2 plays an important role in the pathogenesis of IBD and that blockade of PLD2 may be a new therapeutic target for the management of IBD.

Neutrophils, critical components of innate immune system, are the first line of cellular defense extravasating into inflammatory tissue in response to invading of pathogens and functions as essential mediators in many diseases [37]. Patients with congenital neutrophil disorders are more susceptible to severe infection of bacteria and fungi, suggesting that neutrophils are indispensable in protecting the host from microbes. In patients with chronic granulomatous disease, neutrophils are defective in the production of ROS due to the dysfunction of NADPH oxidase, which culminates in the recurrent infection and life-span shortening and further underscores the importance of neutrophils [38]. In mouse sepsis models, when mice lack some antimicrobial capacity (e.g., degranulation), mice die quickly after the exposure to sepsis [39]. These studies highlight the indispensable role of neutrophils in antimicrobial defense in acute inflammation. However, what does the role of neutrophils in intestinal inflammation of IBD? In DSS-induced mouse colitis, intestinal inflammation has been observed to be exacerbated after the depletion of neutrophils by administration of anti-Gr1 antibodies, indicating a beneficial role of neutrophils in colitis [40–42]. Moreover, neutrophils have been shown to promote the production of several growth factors (e.g., vascular endothelial growth factor), proresolution lipid (e.g., lipoxins, resolvins, and protectins), and anti-inflammatory molecule (e.g., lipoxin A4), contributing to resolution of mucosal inflammation [11]. Interestingly, treatment with lipoxin A4 has been found to ameliorate DSS-induced intestinal inflammation [43]. Furthermore, previous study has confirmed that neutrophils can promote epithelial repairing and mucosal healing through the production of IL-22 [44]. In the current study, our data also demonstrated that percentage and numbers of neutrophils were significantly increased in peripheral blood and inflamed mucosa from patients with IBD, further proving the critical role of neutrophils in the pathogenesis of IBD.

PLD2 is an enzyme that catalyzes the conversion of membrane PC to choline and PA. PLD2 is expressed in nearly all types of leukocytes and has been associated with phagocytosis, degranulation, microbial killing, and leukocyte maturation [20]. Di Fulvio and Gomez-Cambronero found that the expression of PLD2 increased during neutrophil granulocytic differentiation [45]. In another study of sepsis [18], Lee et al. found that PLD2 deficiency not only increases survival but also decreases vital organ damage during experimental sepsis. Production of proinflammatory cytokines (e.g., TNF, IL-17, and IL-23) and cellular apoptosis in kidney and liver are markedly decreased in PLD2^−/−^ mice. PLD2^−/−^ neutrophils significantly protect wild-type mice against sepsis-induced death through the increased formation of NET [18]. In our study, we demonstrated that PLD2 expression was highly increased in peripheral blood and inflamed mucosa of IBD patients and was mainly expressed in neutrophils. Moreover, inhibition of PLD2 ameliorated intestinal mucosal inflammation and reduced the mortality after exposure to DSS. Therefore, PLD2 plays an important role in mucosal inflammation of IBD.

Chemotaxis is one important feature of neutrophils in regulating inflammation [46]. Impairment of chemotaxis has been reported in a wide variety of diseases associated with increased susceptibility to infection. In patients with chronic kidney disease (CKD), FGF23, an endocrine hormone that can regulates phosphorus homeostasis, inhibits the activation and recruitment of neutrophils, and FGF23 neutralization in mouse CKD models restore leukocyte recruitment and host defense [47]. Evidences have been proven that MAPK p38 signaling pathway is required for the recruitment of neutrophils to sites of inflammation and that aberrant p38 signaling in neutrophils aggravates the inflammation in acute lung injury and life-threatening acute respiratory distress syndrome [48]. Moreover, other studies have confirmed that the interaction between neutrophil transepithelial migration and epithelial-expressed ICAM-1 can regulate epithelial homeostasis and promote intestinal mucosal wound healing [49]. These data suggest that neutrophil migration plays a beneficial role in the protection of inflammation. In this study, we found that blockade of PLD2 with selective inhibitor could ameliorate DSS-induced colitis in mice and promote neutrophil mobilization from the bone marrow into intestinal mucosa. CXCR2, a chemokine receptor expressed in neutrophils, mediates neutrophils mobilization from the bone marrow and peripheral blood to the sites of inflammation under the stimulation of its ligand (CXCL1 and CXCL2). In a study of sepsis, IL-33 can reverse the decreased expression of CXCR2 in neutrophils through the inhibition of GRK2 [35, 36]. During viral infection, IFN can suppress CXCR2-mediated neutrophil recruitment into the sensory ganglia [50]. In experimental sepsis model, PLD2 deficiency protects wild-type mice against sepsis-induced death by enhancing neutrophil recruitment. A CXCR2-selective antagonist (SB225002) abolishes the protection conferred by PLD2 deficiency during experimental sepsis, suggesting that enhanced CXCR2 expression in neutrophils promotes survival in PLD2^−/−^ mice [18]. In our study, we found that blockade of PLD2 could promote neutrophil recruitment through upregulating CXCR2 in a GRK2-dependent manner. However, we also found that PLD2 expression was increased in peripheral blood and inflamed mucosa of IBD patients, the mechanisms whereby increased infiltration of neutrophils may be also attributed to other molecules (e.g., TRPC6, IL-18, IL-8, Lewis X, and MAPK p38) at different stages of intestinal mucosal inflammation [51–54]. Moreover, when neutrophils are recruited into inflamed mucosa, they may be engaged in complex bidirectional interactions with epithelium, macrophages, dendritic cells, natural killer cells, and T lymphocytes, which further modulate neutrophil migration at advanced stage of intestinal inflammation [11]. Since these molecules and immune cells are involved in intestinal inflammatory response, regulation of neutrophil migration is a complicate network, and the increased numbers of neutrophils in peripheral blood and inflamed mucosa are the result of mutual modulation of these molecules and immune cells. In our study, PLD2 blockade was the only variable* in vitro*, which could account for the fact that increased neutrophil migration was the result of PLD2 blockade. Therefore, we concluded that blockade of PLD2 improves neutrophil migration.

In summary, our data indicate that PLD2 is a novel indispensable regulator in the pathogenesis of IBD by inhibiting neutrophil migration through downregulating CXCR2 expression. Blockade of PLD2 could ameliorate intestinal mucosal inflammation induced by DSS in mice. Our study has shed a new light on elucidating the role of PLD2 in the pathogenesis of IBD, and blockade of PLD2 may be a new therapeutic target for the management of IBD.

## Supplementary Material

In the supplementary materials, we examined neutrophil profiles in IBD patients. We observed that percentage and absolute numbers of neutrophils were increased in peripheral blood of IBD patients, and neutrophil expression was enhanced in inflamed mucosa of IBD patients. Moreover, PLD2 expression was markedly increased in inflamed mucosa and bone marrow-derived neutrophils in mice with DSS-induced colitis. In addition, information about patients with IBD and healthy donors were listed in supplementary table 1.

## Figures and Tables

**Table 1:** The primers using in qRT-PCR analysis.

Gene	Species	DNA sequence (sense 5′-3′)	DNA sequence (antisense 5′-3′)
PLD2	Human	CAGATGGAGTCCGATGAGGTG	CCGCTGGTATATCTTTCGGTG
IL-17A	Mouse	TTTAACTCCCTTGGCGCAAAA	CTTTCCCTCCGCATTGACAC
IFN-*γ*	Mouse	ATGAACGCTACACACTGCATC	CCATCCTTTTGCCAGTTCCTC
IL-1*β*	Mouse	TTCAGGCAGGCAGTATCACTC	GAAGGTCCACGGGAAAGACAC
IL-10	Mouse	GCTCTTACTGACTGGCATGAG	CGCAGCTCTAGGAGCATGTG
CXCR2	Mouse	TGTCTGGGCTGCATCTAAAGT	AGGTAACCTCCTTCACGTATGAG
GRK2	Mouse	AGCCCTTGGTGGAGTTCTAC	CCCCTCGGAGGTTCTGACA
PLD2	Mouse	GTGGTGGGCACCGAAAGATAC	CATGCGTCAAGCGAACAGAA
GAPDH	Human	CTGGGCTACACTGAGCACC	AAGTGGTCGTTGAGGGCAATG
GAPDH	Mouse	AGGTCGGTGTGAACGGATTTG	GGGGTCGTTGATGGCAACA
