# Supplementary material for: Blockade of PLD2 Ameliorates Intestinal Mucosal Inflammation of Inflammatory Bowel Disease
Source: Mediators Inflamm. 2016 Sep 18;2016:2543070. doi: 10.1155/2016/2543070 (PMC5046040; doi:10.1155/2016/2543070)
Supplement: Supplementary file 1 — In the supplementary materials, we examined neutrophil profiles in IBD patients. We observed that percentage and absolute numbers of neutrophils were increased in peripheral blood of IBD patients, and neutrophil expression was enhanced in inflamed mucosa of IBD patients. Moreover, PLD2 expression was markedly increased in inflamed mucosa and bone marrow-derived neutrophils in mice with DSS-induced colitis. In addition, information about patients with IBD and healthy donors were listed in supplementary table 1. [file 2543070.f1.docx]

**Supplementary Figure 1**


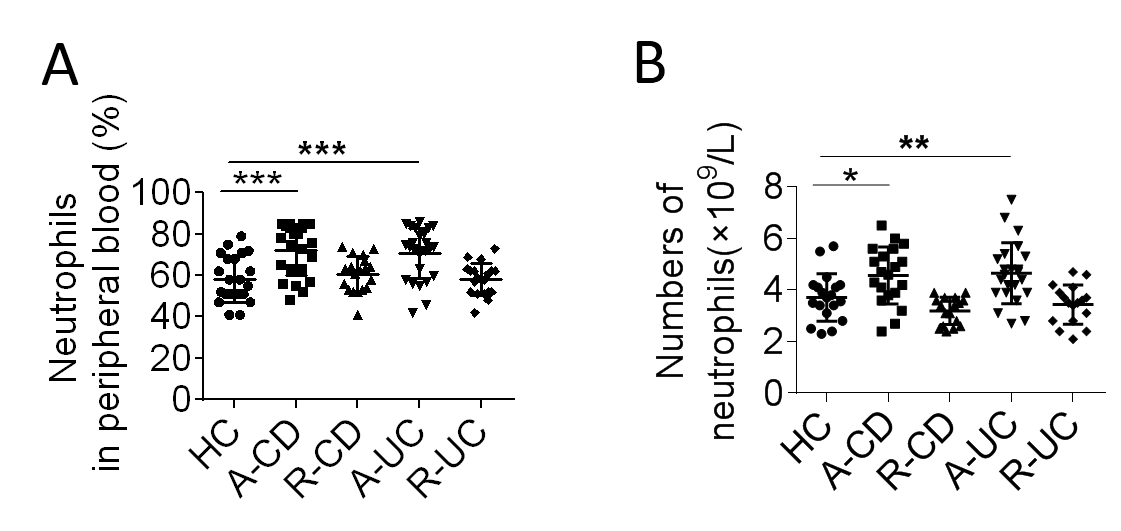


**Supplementary Figure 1 Percentage and numbers of neutrophils are increased in peripheral blood from patients with IBD.** Neutrophils in peripheral blood were analyzed from patients with A-CD (n = 23), R-CD (n = 20), A-UC (n = 26), R-UC (n = 20), and HC (n = 22). Percentage of neutrophils (A) and absolute numbers of neutrophils (B) in peripheral blood in patients with IBD were analyzed. * *p* < 0.05, ** *p* < 0.01, *** *p* < 0.01.

**Supplementary Figure 2**


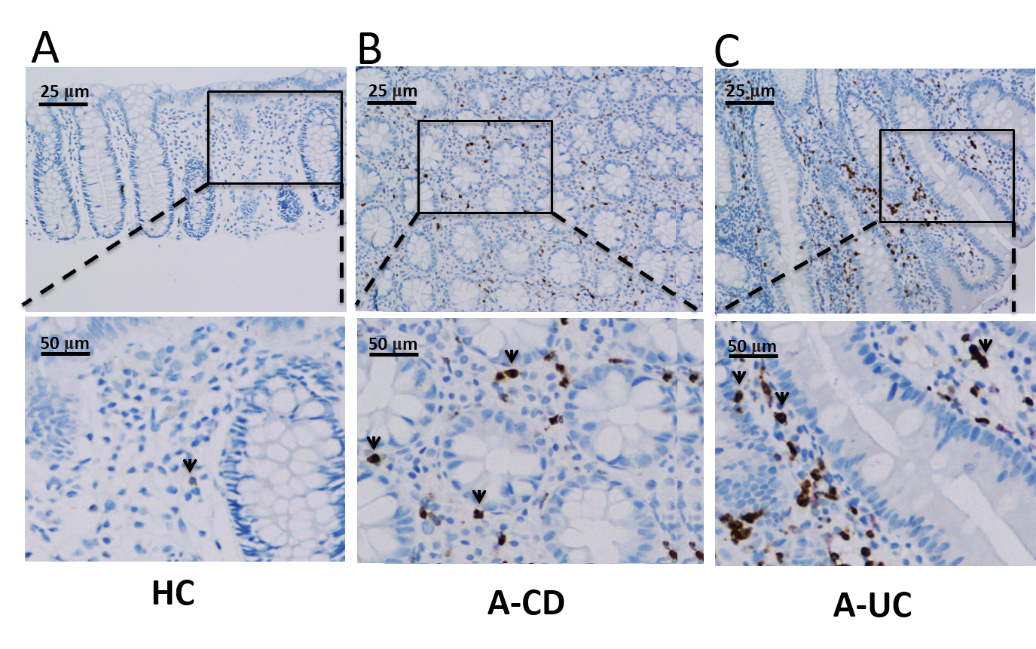


**Supplementary Figure 2 CD66b is highly expressed in inflamed mucosa from patients with IBD.** Representative images of immunohistochemical staining of CD66b expression in normal mucosa from healthy controls (HC, A), and inflamed colon from patients with A-CD (B) or A-UC (C). Original magnifications ×200 (top), and ×400 (bottom).

**Supplementary Figure 3**


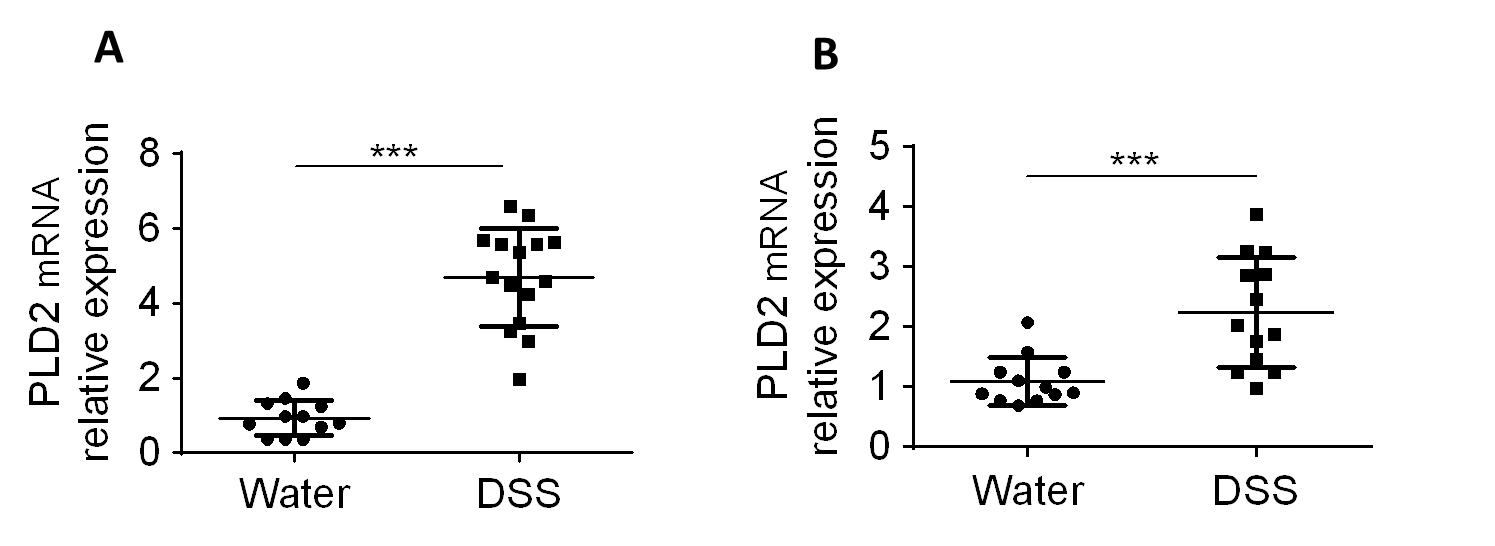


**Supplementary Figure 3 Expression of PLD2 is significantly increased in inflamed mucosa and neutrophils from bone marrow in DSS-induced colitis model in mice.** (A) Acute colitis in wild-type C57BL/6 mice was induced by 2.5% DSS as described. Colonic tissues were obtained from DSS-induced colitis mice (n = 15) and control mice (n = 12). The mRNA levels of PLD2 were detected by qRT-PCR. * *p* < 0.05, ** *p* <0.01, *** *p* < 0.001 versus controls. (B) Neutrophils were isolated from the bone marrow of DSS-induced colitis mice (n = 13) and control mice (n = 12). The mRNA levels of PLD2 were detected by qRT-PCR. * *p* < 0.05, ** *p* < 0.01, *** *p* < 0.01 versus controls.

Supplementary Table 1. Clinical characteristics of patients with IBD

|  | Blood samples | | |  | Biopsy samples | | |
| --- | --- | --- | --- | --- | --- | --- | --- |
|  | HC | CD (A/R) | UC (A/R) |  | HC | CD (A/R) | UC (A/R) |
| Number of patients | 28 | 44 (25/19) | 41 (20/21) |  | 18 | 48 (21/27) | 52 (26/26) |
| Age (y) | 30.45±12.5 | 26.45±8.45 | 39.54±18.54 |  | 35.46±8.56 | 28.16±7.45 | 41.54±11.68 |
| Gender |  |  |  |  |  |  |  |
| Male | 15 | 24 (13/11) | 17 (10/7) |  | 9 | 23 (13/10) | 30 (14/16) |
| Female | 13 | 20 (12/8) | 24 (10/14) |  | 9 | 25 (8/17) | 22 (12/10) |
| Disease duration (month) |  | 36.5±19.5 | 47.5±23.6 |  |  | 38.6±16.6 | 44.8±15.5 |
| Current therapy |  |  |  |  |  |  |  |
| 5-aminosalicylates |  | 34 (20/14) | 32 (13/19) |  |  | 37 (15/22) | 36(24/12) |
| Immunosuppressants |  | 0 | 0 |  |  | 0 | 0 |
| Biologics |  | 0 | 0 |  |  | 0 | 0 |
| Nutritional therapy |  | 5 (5/0) | 7(7/0) |  |  | 6 (6/0) | 2 (2/0) |
| Disease extent (UC)^*^ |  |  |  |  |  |  |  |
| E1 |  |  | 3 (2/1) |  |  |  | 9 (3/6) |
| E2 |  |  | 20 (12/8) |  |  |  | 13 (7/6) |
| E3 |  |  | 18 (6/12) |  |  |  | 30 (16/14) |
| Disease location (CD)^*^ |  |  |  |  |  |  |  |
| L1 |  | 6 (4/2) |  |  |  | 8 (3/5) |  |
| L2 |  | 15 (9/6) |  |  |  | 12 (4/8) |  |
| L3 |  | 23 (12/11) |  |  |  | 28 (14/14) |  |
| L4 |  | 0 |  |  |  | 0 |  |
| CRP (mg/L) |  | 30.5±12.5 | 36.7±14.5 |  |  | 30.8±11.8 | 32.4±13.2 |

^*^According to the Montreal classification system. A/R: Active/Remission
